# Chromosomal Behavior during Meiosis in the Progeny of *Triticum timopheevii* × Hexaploid Wild Oat

**DOI:** 10.1371/journal.pone.0126398

**Published:** 2015-05-07

**Authors:** Hongzhou An, Mei Hu, Pengfei Li, Guangdong Geng, Qingqin Zhang, Suqin Zhang

**Affiliations:** 1 College of Agriculture, Guizhou University, Guiyang, Guizhou, China; 2 Guizhou Subcenter of National Wheat Improvement Center, Guiyang, Guizhou, China; Institute of Genetics and Developmental Biology, CHINA

## Abstract

The meiotic behavior of pollen mother cells (PMCs) of the F_2_ and F_3_ progeny from *Triticum timopheevii* × hexaploid wild oat was investigated by cytological analysis and sequential C-banding-genomic *in situ* hybridization (GISH) in the present study. A cytological analysis showed that the chromosome numbers of the F_2_ and F_3_ progeny ranged from 28 to 41. A large number of univalents, lagging chromosomes, chromosome bridges and micronuclei were found at the metaphase I, anaphase I, anaphase II and tetrad stages in the F_2_ and F_3_ progeny. The averages of univalents were 3.50 and 2.73 per cell, and those of lagging chromosomes were 3.37 and 1.87 in the F_2_ and F_3_ progeny, respectively. The PMC meiotic indices of the F_2_ and F_3_ progeny were 12.22 and 20.34, respectively, indicating considerable genetic instability. A sequential C-banding-GISH analysis revealed that some chromosomes and fragments from the hexaploid wild oat were detected at metaphase I and anaphase I in the progeny, showing that the progeny were of true intergeneric hybrid origin. The alien chromosomes 6A, 7A, 3C and 2D were lost during transmission from F_2_ to F_3_. In addition, partial *T*. *timopheevii* chromosomes appeared in the form of univalents or lagging chromosomes, which might result from large genome differences between the parents, and the wild oat chromosome introgression interfered with the wheat homologues’ normally pairing.

## Introduction

Wide crosses in the *Triticeae* tribe have been performed and studied for over 100 years [1]. Wild relatives of common wheat, *Triticum aestivum*, and related species are an important genetic reservoir for the improvement of their cultivated counterparts [2]. Several useful traits, including resistance to diseases and pests, stress and salt tolerance, and winter hardiness have been transferred from these species to wheat [3]. To date, the intergeneric hybridizations have been successful between *Triticum* and *Secale* [1,4–5], *Elytrigia* [6–7], *Aegilops* [8–9], *Haynaldia* [10–11], *Hordeum* [12–13], *Roegneria* [14–15], *Elymus* [16–17], *Leymus* [18–19], *Agropyron* [20–21] and *Zea* [22–23].

*T*. *timopheevii* Zhuk. (A^t^A^t^GG, 2n = 28) is a tetraploid wheat and species of *Triticum* L. that occupies a unique position in the genus *Triticum* [24]. It shows high levels of resistance to several diseases, such as leaf and stripe rusts [25–26], and powdery mildew [26]. Therefore, it is selected as an important resistance source for wheat breeding. In hybrid breeding programs the cytoplasmic male sterile (CMS) trait is of great value to plant breeders because laborious hand-emasculation is avoided. *T*. *timopheevii* has attracted the attention of wheat breeders owing to the presence of cytoplasmic male sterility and fertility restorer factors [27]. In addition, the high-molecular-weight (HMW) subunits of glutenin are particularly important for wheat gluten and dough elasticity [28]. Wan et al. [29] isolated three novel genes encoding HMW subunits from *T*. *timopheevii*.

Wild oats belong to the genus of *Avena*, which possess rich nutrients, such as protein and lysine [30–31]. Meanwhile, *Avena fatua* has contributed arid-region adaptation, rapid growth, earliness and complex resistances to diseases (immune or high resistances to powdery mildew, rust and scab of wheat) [31–33]. Hexaploid *A*. *fatua* L. (AACCDD) is a particularly useful source because it is a hexaploid species. Wild oats have the potential to serve as a genetic reservoir for wheat improvement. However, the hybridization between *T*. *timopheevii* and wild oats has seldom been reported. In the present study, the hybrids *T*. *timopheevii* × hexaploid *A*. *fatua* L. were bred to transfer useful traits from oat to wheat, which would be used as a bridge for breeding bread wheat.

Each generation in a sexually reproducing organism passes through the bottleneck of meiosis, which is the specialized cell division that gives rise to haploid reproductive cells. In the present study, we report the chromosome behavior at meiosis of the progeny from *T*. *timopheevii* × hexaploid *A*. *fatua* L. using a cytological analysis and the sequential C-banding-GISH (genomic *in situ* hybridization) technique. Its main objective was to understand chromosomic meiotic behavior and alien chromosome characterization in the *Triticum* background to improve the efficacy of its use. Results of this study may be helpful for enriching genetic and breeding germplasm, and investigating the origin and evolution of wheat.

## Materials and Methods

### Plant materials

*T*. *timopheevii* (A^t^A^t^GG) and *A*. *fatua* L. (AACCDD, which was originally collected in Portugal) are pure breeding lines kept at the Guizhou Subcenter of National Wheat Improvement Center, China. The experiments were conducted at Guizhou University (Guiyang, China) from 2010 to 2013. *T*. *timopheevii* (♀) was crossed with the hexaploid wild oat (♂), and the F_1_ plants were self-pollinated to generate the F_2_ and F_3_ progeny. The F_2_ and F_3_ progeny (25 plants/each generation) were randomly selected for the present study.

### Methods

#### Meiotic analysis

Appropriate young spikes (with flag leaves of about 3–4 cm in length) were excised in the morning (7:30–10:30 AM). The anthers were taken out and fixed in 3:1 ethanol: glacial acetic acid for 2–7 d, squashed in a drop of 45% acetic acid with 1% carmine, and examined using phase-contrast optics. Images of intact cells were then captured using a Spot CCD camera (Micropublisher 5.0; QImaging, Surrey, BC Canada). The meiotic index (mi) was defined as the percentage of normal quartets recorded. Normal tetrads were considered as those with four equal-sized cells. Meiotic indices were calculated from 150 tetrads per plant according to Sapra and Heyne [34] formula: mi = (number of normal tetrads/total of tetrads) × 100. Stable inheritance was indicated by a value of 90–100 mi.

#### Sequential C-banding-GISH analysis

The C-banding was performed essentially as described by Jellen et al. [35]. Total genomic DNA from *T*. *timopheevii* and the hexaploid wild oat was extracted using the CTAB method following the phenol-chloroform method described by Zheng [36]. Genomic DNA of the wild oat was used as a probe and labeled with digoxigenin-11-dUTP (DIG-Nick Translation Mix; Roche Applied Science, Indianapolis, IN USA) according to the manufacturer’s instructions. Sheared *T*. *timopheevii* genomic DNA was used as blocking DNA at a ratio of 80:1 (block DNA: probe DNA). The GISH procedure was carried out as described by Zheng [36] with minor modifications in signal detection. After overnight hybridization at 37°C in the hybridization system of ThermoBrite version 2.12 (Abbott, Molecular, Inc, Des Plaines, IL USA), the slides were washed at 42°C in 2×SSC for 10 min, followed by washing with 2×SSC and 1×TNT for 5 min each at room temperature. Digoxigenin-labeled DNA was detected with anti-digoxigenin-rhodamine Fab fragments (Roche Applied Science) for 1 h at 37°C, and then slides were washed for 3×5 min in 1×TNT at room temperature. The slides were mounted in Vectashield antifade solution (Vector Laboratories, Inc., Burlingame, CA USA) containing 0.2 μg/mL 4′-6-diamino-2-phenylindole (DAPI; Roche Applied Science). A fluorescence microscope (BX60 Olympus Corp., Tokyo, Japan) fitted with a Spot CCD camera was used to capture hybridization signals with separate filters for detecting DAPI and rhodamine signals. The images were compiled with CellSens Vers.1.5 Imaging software (Olympus Corp.).

## Results

### Chromosome meiotic behavior in the progeny

**Fig 1:**
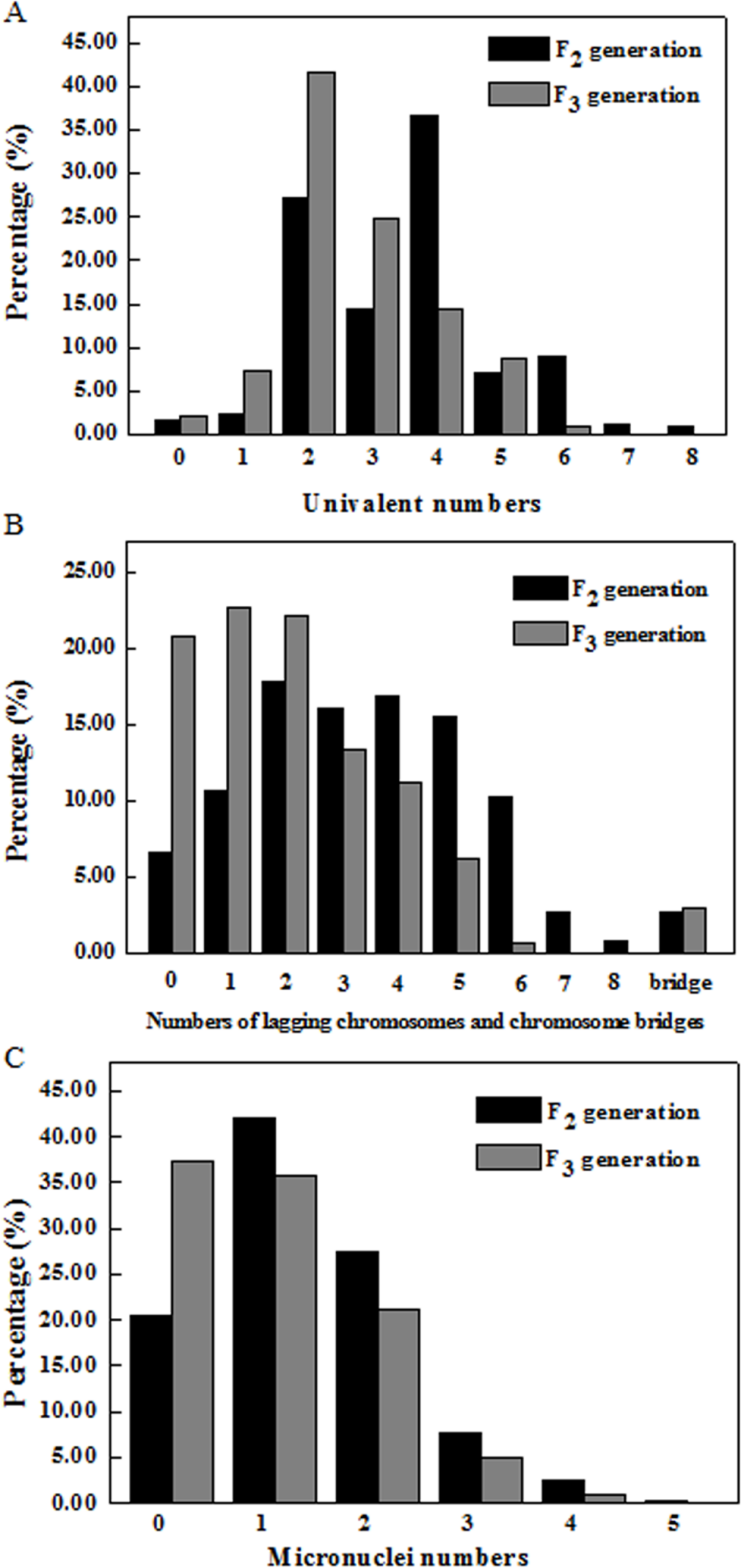
The percentage of irregular chromosomes at metaphase I, anaphase I and telophase I in the pollen mother cells’ (PMCs) in the *Triticum timopheevii* × hexaploid wild oat F_2_ and F_3_ generations, respectively. A, The x-axis shows the numbers of univalents in each PMC at metaphase I. B, The x-axis shows the numbers of lagging chromosomes and chromosome bridges in each PMC at anaphase I. C, The x-axis shows the numbers of micronuclei in each PMC at telophase I.

**Fig 2:**
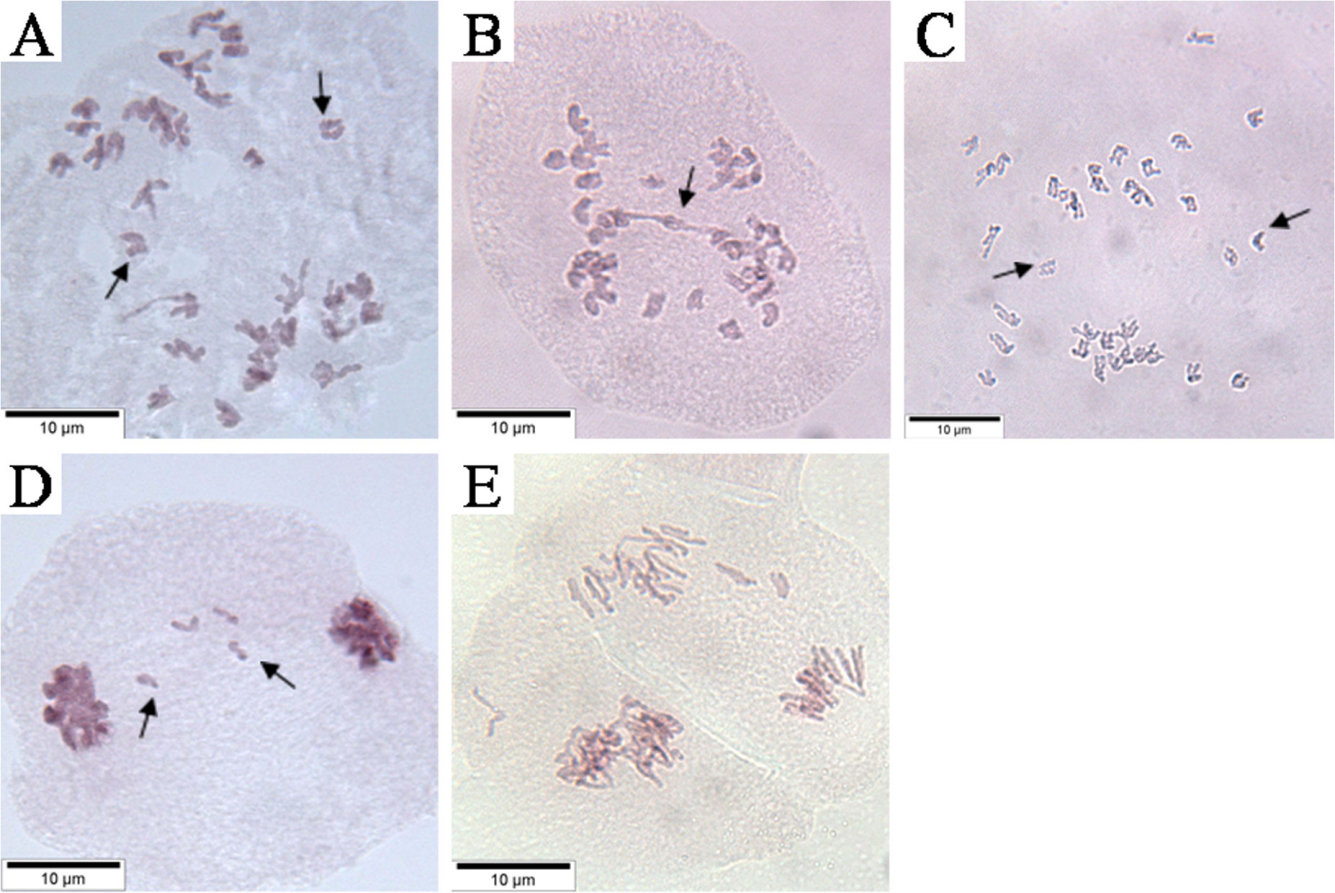
The pollen mother cells’ (PMCs) meiotic behavior in some *Triticum timopheevii* × hexaploid wild oat F_2_ plants. A and B, Anaphase I in the plant TPF2-1. Arrows show lagging chromosomes (A) and chromosome bridge (B). C, Anaphase I in the plant TPF2-2. Arrows show lagging chromosomes. D, Telophase I in the plant TPF2-1. Arrows show lagging univalent. E, Anaphase II in the plant TPF2-2, showing daughter cells from the first division were non-synchronous. Scale bar = 10 μm.

The meiotic behavior of the F_2_ and F_3_ progeny from *T*. *timopheevii* × hexaploid *A*. *fatua* L. was studied in detail. For the F_2_ plants, the chromosome numbers were 28–41 (S1 Table). The frequency of univalents ranged from 0–8 in various cells (Fig 1A). There were mostly four, two and three univalents per cell in the F_2_ plants at frequencies of 36.67%, 27.11% and 14.44%, respectively (Fig 1A, S2 Table). At anaphase I, there were lagging chromosomes (0–8) (Fig 1B, S1 Table) and chromosome bridges (2.67%) (Fig 1B, S2 Table) in the F_2_ plants. Two lagging chromosomes (17.78%) appeared the most often, followed by four (16.89%) and three (16.00%) lagging chromosomes (Fig 1B, S2 Table). Two types of lagging chromosomes were found: one in which the centromeres of sisters did not separate, so two sister chromosomes moved together toward a pole (Fig 2A–2C) and one in which the centromeres of the sisters separated, and the two sister chromosomes separated toward the respective poles (Fig 2D). Some lagging chromosomes failed to enter the polar region where the other chromosomes started to compact and then formed micronuclei at telophase I (Fig 1C, S1 Table). A total of 79.56% of the cells had micronuclei, and the percentage of 1 or 2 micronuclei in a cell was high, reaching up to 69.33% in the F_2_ plants (Fig 1C, S2 Table). The numbers and sizes of the micronuclei could be different at the two poles due to an abnormal first meiosis, so some chromosomes segregated asymmetrically into two daughter cells.

The chromosome numbers were 28–36 in the F_3_ strains (S1 Table), and the maximum was less than that of the F_2_ generation. The averages for the univalents, lagging chromosomes and the micronuclei in the F_3_ generation were less those of the F_2_ generation (S1 Table). The frequency of univalents ranged from 0–6 in various cells at metaphase I in the F_3_ strains (Fig 1A). There were a large number of two, three and four univalents per cell at frequencies of 41.67%, 24.83% and 14.50%, respectively (Fig 1A, S2 Table). At anaphase I, there were lagging chromosomes (0–6) (Fig 1B, S1 Table) and chromosome bridges (3.00%; Fig 1B) in the F_3_ strains. One lagging chromosomes (22.67%) appeared the most often, followed by two (22.17%) and zero (20.83%) lagging chromosomes in the F_3_ strains (Fig 1B, S2 Table). A total of 62.66% of the cells had micronuclei, and the percentage of 1 or 2 micronuclei in a cell was high, reaching up to 56.84% in the F_3_ strains (Fig 1C, S2 Table). The wide genetic distance and genome differences between the two parents may lead to intergenomic conflicts and chromosome pairing irregularly in the progeny.

At anaphase II, some daughter cells from the first division were obviously non-asynchronous (Fig 2E); one might have reached anaphase II while the other was still in metaphase II in the F_2_ plant. The timing mechanism may have failed as a result of genome interactions in the *T*. *timopheevii* × hexaploid wild oat hybrid progeny.

**Fig 3:**
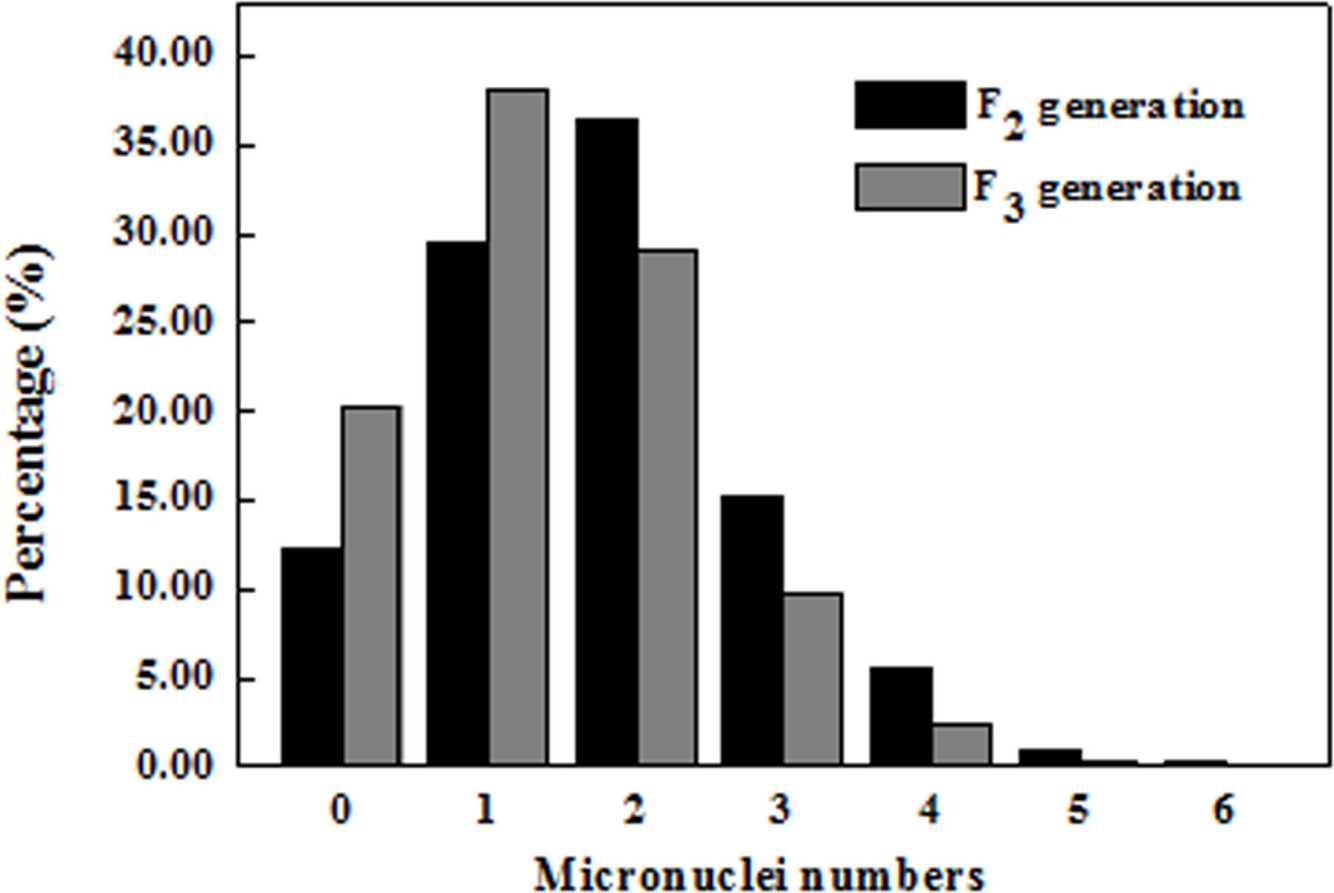
The percentage of pollen mother cells’ (PMCs) with micronuclei in the *Triticum timopheevii* × hexaploid wild oat F_2_ and F_3_ generations at telophase II.

At telophase II, the majority of cells had various numbers and sizes of micronuclei in the F_2_ and F_3_ progeny (Fig 3). The most predominant number of micronuclei in a cell was two, next was one, and then three in the F_2_ plants, which was more than that of the F_3_ strains generally. For the F_3_ strains, the abnormal cell average (119.50; S3 Table) was lower than that (131.67) of the F_2_ plants. The averages of the meiotic indices (mi) were 12.22 and 20.34 for the generations F_2_ and F_3_, respectively (S1 Table), indicating that they were quite genetically unstable.

In a comprehensive comparison of the meiotic processes, the F_2_ and F_3_ progeny showed consistent abnormal behaviors, but the degree of abnormality in the F_3_ progeny was lower than that in the F_2_ progeny. The wide genetic distance and great genome differences between the two parents might lead to the PMCs’ abnormal meiotic behavior in the progeny.

### Sequential C-banding-GISH analysis

**Fig 4:**
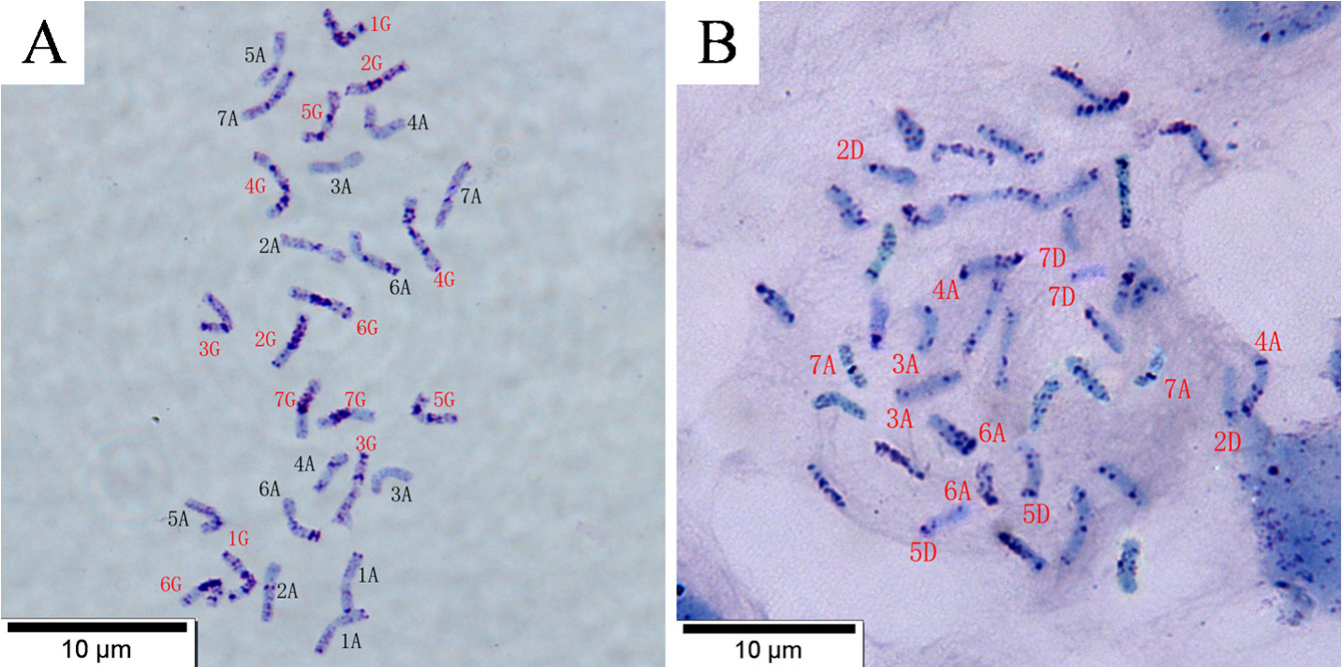
C-banding pattern of root tip cells in *T*. *timopheevii* (A) and the hexaploid *Avena fatua* L. (B). Scale bar = 10 μm.

The maternal genome had 28 chromosomes and a genome designation of A^t^A^t^GG (Fig 4A). The large amounts of heterochromatin in the centromeric and interstitial regions belonged to the G genome. The A genome had less heterochromatin, which was located mainly at the centromeres and telomeres. These results were in agreement with previous reports [37–38]. The C-banded karyotype of the hexaploid wild oat was composed of dark-staining C-genome chromosomes and light-staining A and D genomes (Fig 4B). The C-banding patterns were similar to those described in previous reports on cultivated oats or wild oats [35,39–40]. The most striking C-banding feature was that the staining speed and intensity of chromosomes originating from the two parents were distinctly different in the progeny; the chromosomes from *T*. *timopheevii* were darkly stained, while those from the wild oat were only lightly stained.

**Fig 5:**
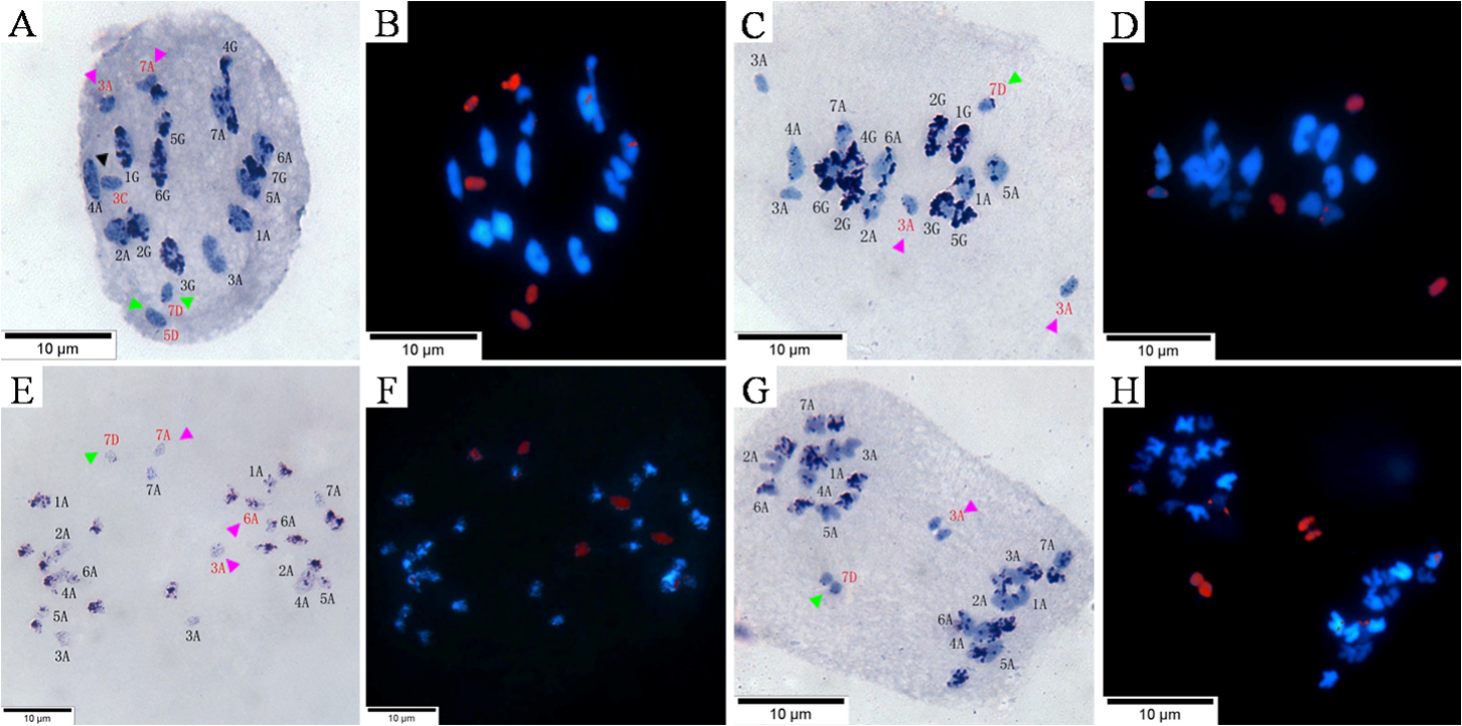
Sequential C-banding- genomic *in situ* hybridization (GISH) analysis of the pollen mother cells’ (PMCs) of the *Triticum timopheevii* × hexaploid wild oat F_2_ and F_3_ progeny. A and B, Sequential C-banding-GISH analysis of the PMCs in an F_**2**_ plant at metaphase I. C and D, Sequential C-banding-GISH analysis of the PMCs in an F_**3**_ plant at metaphase I. E and F, Sequential C-banding-GISH analysis of the PMCs in an F_**2**_ plant at anaphase I. G and H, Sequential C-banding-GISH analysis of the PMCs in an F_**3**_ plant at anaphase I. Arrows show the chromosomes from the hexaploid *A*. *fatua* L.: red, black and green arrows show A-, C- and D- genome chromosomes, respectively. The chromosomes were counterstained with DAPI (blue), and hybridization sites are in red. Scale bar = 10 μm.

Sequential C-banding-GISH analysis was applied to investigate the PMCs of some F_2_ and F_3_ progeny at metaphase I and anaphase I. The chromosome configuration was 2n = 14 II+5 I at metaphase I in the cell of an F_2_ plant (Fig 5A and 5B). All five univalents yielded red wild oat-specific hybridization signals, which indicates a wild oat origin (Fig 5B). Their C-bands were light, and similar to those of its male parent (Fig 5A). Three of five univalents showed red wild oat-specific hybridization signals in the cell of an F_3_ plant (Fig 5D), which had C-bands similar to the chromosomes 10, 10 and 18 [35], and the chromosomes 3A, 3A and 7D (Fig 5C) [39]. Some dotted fragments were found on some chromosomes from *T*. *timopheevii* in both cells.

Three of five alien chromosomes were lagging in the cell of an F_2_ plant at anaphase I (Fig 5G and 5H) in which C-bandings were similar to the 3A, 7A and 3D chromosomes [39]. The other two alien chromosomes moved to the same pole. One of them was similar to the 6A chromosome [39], and the other (with the black arrow) should belong to the C-genome based on the dark staining. The chromosomes 3A, 6G and 7A originating from *T*. *timopheevii* were lagging, indicating the chromatin introgression of the wild oat interfered with the original genetic balance leading to the irregular behavior. Two alien chromosomes were lagging in the cell of an F_3_ plant (Fig 5G and 5H) in which C-bandings were similar to the 3A and 7D chromosomes [39].

Chromosomes 3A, 4A, 6A, 7A, 3C, 2D, 5D and 7D from the wild oat were detected in the F_2_ progeny (Table 1), among which chromosomes 3A, 4A, 7A and 7D appeared most frequently. The alien chromosomes 6A, 7A, 3C and 2D were lost during transmission from F_2_ to F_3_. However, chromosomes 3A, 4A, 5D and 7D were more stable and were transmissible to the next generation, indicating that these chromosomes might contain chromatin that is more similar to that of *Triticum timopheevii*. All of the *T*. *timopheevii* chromosomes in the F_2_ progeny containing fragments from the wild oat seemed to be transmitted to the F_3_ progeny, indicating that these short fragments are more stable than the intact wild oat chromosomes during the F_2_ to F_3_ transmission.

**Table 1:** The detected alien chromatin in the F_2_ and F_3_ progeny.

Generations	Intact wild oat chromosomes in the progeny	The *T*. *timopheevii* chromosomes containing fragments from the wild oat in the progeny
**F**_**2**_ **generation**	3A, 4A, 6A, 7A, 3C, 2D, 5D, 7D	3A, 6A, 7A, 4G, 5G, 6G
**F**_**3**_ **generation**	3A, 4A, 5D, 7D	3A, 6A, 7A, 4G, 5G, 6G
The alien chromatin lost during the transmission from the F_2_ to F_3_	6A, 7A, 3C, 2D	None

The F_2_ and F_3_ progeny from *Triticum timopheevii* × hexaploid wild oat were aneuploid plants with complete *T*. *timopheevii* chromosomes and different numbers of retained wild oat chromosomes. The aneuploidy might have occurred through non-disjunction. Indeed, non-disjunction of chromosomes was observed in the progeny cells (Fig 1A–1C). The chromosome numbers of the progeny ranged from 28 to 41 in this study, so they were identified as *T*. *timopheevii × A*. *fatua* L. asymmetric hybrid progeny. Parental genomes were spatially separated within the progeny nuclei, and the wild oat chromatin (in red) occupied a predominantly peripheral position at metaphase I. In addition, most wild oat chromosomes tended to remain near the equatorial plate, apparently lagging, while the wheat chromosomes moved to the spindle poles during anaphase I (Fig 5E–5H). The facts suggested that the wild oat chromosomes were delayed in their movement toward the spindle pole compared with the wheat chromosomes at anaphase.

## Discussion

In recent years, wide crosses between crop plants and their wild relatives have attracted significant attention as sources of desirable characteristics for the genetic improvement of crops. Sometimes it is imperative that genetic material from wide hybrids be developed and exploited in breeding programs [41]. Our research group began making wide crosses between *Triticum* (common wheat, emmer, durum and einkorn) and *A*. *fatua* L. (diploid, tetraploid and hexaploid wild oats) in 1978, and the hybrids *T*. *timopheevii* × hexaploid wild oat were obtained in the study. It is quite difficult for the two species to cross naturally, because their flowering behavior is quite different. The complex good strains were selected from their hybrid progeny with high yield and quality, and multiple disease resistances. Over the past 2 years, take-all disease and yellow dwarf viruses in wheat were destructive in Guiyang and other regions of China, but most of the progeny from *A*. *fatua* L. showed a high level of resistance or immunity to them (unpublished data). The progeny from the wide cross *T*. *timopheevii* × *Avena fatua* L. showed a wide genetic distance from the current cultivated wheat in China [42]. Therefore, the germplasm might be a very valuable new resource for improvement of cultivated wheat.

The chromosome pairing in natural intergeneric hybrids was highly irregular, with 14 to 28 univalents per cell [43]. In this study, a large number of univalents, lagging chromosomes, chromosome bridges and micronuclei were found at the metaphase I, anaphase I, anaphase II and tetrad stages in the F_2_ and F_3_ progeny. The averages of univalents were 3.50 and 2.73 per cell at metaphase I, and those of lagging chromosomes were 3.37 and 1.87 at anaphase I in the F_2_ and F_3_ progeny, respectively. Similar observations had also been reported by Finch and Bennett and Xie et al. [44–45]. In this study, the irregular tetrad frequency of micronuclei at telophase II was less than that of the lagging chromosomes at anaphase I. This may result because, while the majority of lagging chromosomes at anaphase I are detained in the cytoplasm and produce micronuclei during meiosis, some lagging chromosomes failed to produce nuclear envelopes and thus disintegrated, and alien chromosomes were lost during genetic transfer. Abnormalities in the second division were not as prevalent as in the first division, suggesting that the highly abnormal PMCs did not proceed very far. We also found that the alien chromosomes 6A, 7A, 3C and 2D were lost during the transmission from the F_2_ to F_3_ in this study.

Gernand et al. [46] studied the mechanisms underlying selective elimination of the paternal chromosomes during the development of wheat × pearl millet hybrid embryos and found that heterochromatinization and DNA fragmentation of micronucleated pearl millet chromatin is the final step during haploidization. The numerous micronuclei, as a form of alien chromosome elimination, may be related to the malfunction of lagging chromosomes, which failed to enter the newly produced nuclei, and in turn generated micronuclei at telophase I and II [44]. These cytological observations showed that chromosome elimination frequently took place during meiosis. Parental genomes were spatially separated within the hybrid nucleus, and the pearl millet chromatin destined for elimination occupied peripheral interphase positions [46]. A similar phenomenon was found in this study; parental chromosomes were spatially separated within the progeny nucleus, and the wild oat chromatin occupied a predominantly peripheral position at metaphase I.

The most striking feature found was that the staining speed and intensity of the C-banding of progeny chromosomes originating from the two parents were distinctly different: the chromosomes from *T*. *timopheevii* were darkly stained, while those from the wild oat were only lightly stained. The different staining intensities of the heterochromatin between the parental chromosomes may indicate a different degree of chromatin condensation. Some whole chromosomes and short fragments from the wild oat were detected by sequential C-banding-GISH analysis in the progeny. The progeny with additions or translocations, as well as restructured alien chromosomes may supply very valuable new germplasm for wheat improvement and genetics. Most of the lagging chromosomes in the progeny at anaphase I came from the wild oat but a few came from *T*. *timopheevii*, and the behavior appeared obviously abnormal. The wild oat introgression may disturb chromosome behavior in the progeny due to the wide intergeneric hybrid origin.

In this study, some daughter cells from the first division showed noticeable asynchronous cell cycles. For example, one had reached anaphase II while the other was still at metaphase II, which may be attributable to the asynchrony of DNA replication. Assuming that the timing of DNA replication differs between wheat and pearl millet, as reported for the parental genomes of *Nicotiana tabacum* hybrids [47], the asynchrony of DNA replication may lead to the breakage of pearl millet chromosomes. Paternal pearl millet chromatin initially underwent extensive fragmentation immediately prior to haploidization of the maternal genome [46]. Alternatively, a hybridization-mediated genomic shock [48] might trigger a genome-specific activation of mobile elements and thus, cause structural chromosome aberrations as reported for artificial allopolyploids of wheat [49] and *Arabidopsis thaliana* [50]. These perspectives may be related to the dotted wild oat signals in the progeny in this study.

Investigation of the genetic mechanisms of the progeny from the wide cross *T*. *timopheevii* × *Avena fatua* L. is interesting, and, presently, we are investigating it further using integrated methodologies and techniques. When fully understood, it may lead to significant advances in wheat genetics and breeding.

## Supporting Information

S1 Table Chromosome configuration of meiosis in the pollen mother cells’ (PMCs) in *Triticum timopheevii* × hexaploid wild oat F_2_ and F_3_ generations. (PDF)

S2 Table The percentage of irregular chromosomes at metaphase I, anaphase I, telophase I and telophase II in the pollen mother cells’ (PMCs) in the *Triticum timopheevii* × hexaploid wild oat F_2_ and F_3_ generations, respectively. (PDF)

S3 Table Frequency of meiotic abnormalities in the pollen mother cells’ (PMCs) in *Triticum timopheevii* × hexaploid wild oat F_2_ and F_3_ generations. (PDF)
