# Supplementary material for: Chromosomal Behavior during Meiosis in the Progeny of Triticum timopheevii × Hexaploid Wild Oat
Source: PLoS One. 2015 May 7;10(5):e0126398. doi: 10.1371/journal.pone.0126398 (PMC4423983; doi:10.1371/journal.pone.0126398)
Supplement: S1 Table — (PDF) [file pone.0126398.s001.pdf]

Supplementary Table S1. Chromosome configuration of meiosis in the pollen mother cells' (PMCs) in *Triticum timopheevii* × hexaploid wild oat F_2 and F_3 generations

ls	Chromosome number	Metaphase I			Anaphase I	Telophase II	Meiotic
		Rod (average)	Ring(average)	Univalents(average)	Lagging chromosomes(average)	Micronuclei(average)	
on	28-41	0.86-1.10(0.94)	13.27-14.13(13.76)	2.91-4.00(3.50)	1.80-4.73(3.37)	1.51-1.97(1.76)	10.00-16.[illegible]
on	28-36	0.06-0.37(0.29)	13.33-14.25(13.68)	1.74-4.01(2.73)	0.65-3.39(1.87)	0.89-1.77(1.38)	8.67-38.[illegible]
