# Supplementary material for: Chromosomal Behavior during Meiosis in the Progeny of Triticum timopheevii × Hexaploid Wild Oat
Source: PLoS One. 2015 May 7;10(5):e0126398. doi: 10.1371/journal.pone.0126398 (PMC4423983; doi:10.1371/journal.pone.0126398)
Supplement: S2 Table — (PDF) [file pone.0126398.s002.pdf]

Supplementary Table S2. The percentage of irregular chromosomes at metaphase I, anaphase I, telophase I and telophase II in the pollen mother cells' (PMCs) in the *Triticum timopheevii* × hexaploid wild oat F_2 and F_3 generations, respectively.

Number / Percentage (%)		0	1	2	3	4	5	6	7	8	
Univalents at metaphase I	F_2 generation	1.56	2.22	27.11	14.44	36.67	7.11	8.89	1.11	0.89	
	F_3 generation	2.00	7.33	41.67	24.83	14.50	8.67	1.00			
Lagging chromosomes at anaphase I	F_2 generation	6.67	10.66	17.78	16.00	16.89	15.56	10.22	2.67	0.89	
	F_3 generation	20.83	22.67	22.17	13.33	11.17	6.17	0.67			
Chromosome bridge at anaphase I	F_2 generation										2.67
	F_3 generation										3.00
Micronuclei at telophase I	F_2 generation	20.44	42.00	27.33	7.56	2.44	0.22				
	F_3 generation	37.34	35.67	21.17	5.00	0.84					
Micronuclei at telophase II	F_2 generation	12.22	29.55	36.45	15.11	5.55	0.89				
	F_3 generation	20.34	38.00	29.00	9.67	2.50	0.34				
