# Supplementary material for: Chromosomal Behavior during Meiosis in the Progeny of Triticum timopheevii × Hexaploid Wild Oat
Source: PLoS One. 2015 May 7;10(5):e0126398. doi: 10.1371/journal.pone.0126398 (PMC4423983; doi:10.1371/journal.pone.0126398)
Supplement: S3 Table — (PDF) [file pone.0126398.s003.pdf]

Supplementary Table S3. Frequency of meiotic abnormalities in the pollen mother cells' (PMCs) in *Triticum timopheevii* × hexaploid wild oat F_2 and F_3 generations

Stage	No. of PMCs analyzed	No. of abnormal PMCs (average)	
		F_2 generation	F_3 generation
**Metaphase I**	150	147-149(147.67)	143-150(147.00)
**Anaphase I**	150	126-149(140.00)	90-145(118.75)
**Telophase I**	150	111-130(119.33)	70-119(94.00)
**Telophase II**	150	126-134 (131.67)	93-137(119.50)
